# Bone metabolism and bone mineral density in adolescent idiopathic scoliosis

**DOI:** 10.1186/1748-7161-10-S1-P7

**Published:** 2015-01-19

**Authors:** Ko Ishida, Yoichi Aota, Naoto Mitsugi, Tomoyuki Katsuhata, Takayuki Higashi, Katsutaka Yamada, Tomoyuki Saito

**Affiliations:** 1 Yokohama City University Medical Center, Japan; 2 Yokohama Brain and Stroke Center, Japan; 3 Yokohama City University, Japan

## Objective

To characterize bone metabolism in AIS patients using bone metabolism markers.

## Summary of background data

Although osteopenia is often associated with AIS, bone metabolism in this condition has not been assessed.

## Methods

Bone mineral density (BMD) of the lumbar spine and bilateral proximal femurs (dual energy x-ray absorptiometry) and bone metabolism markers {bone formation marker: serum bone alkaline phosphatase (BAP); bone resorption marker: serum tartrate-resistant acid phosphatase serum band 5 (TRAP5b)} were measured in 55 consecutive AIS subjects aged 10 to 18 years-old (mean: 15.6+/-1.7). BMD, body mass index (BMI), and age of menarche were compared between subjects with normal and high values of TRAP5b.

## Results

Nineteen subjects (34%) had osteopenia and 17 subjects (31%) had osteoporosis. In 51 AIS subjects (93%), values for BAP were within normal range, while 33 subjects (60%) had high values for TRAP5b. Subjects with high values for TRAP5b had BMDs of the lumbar spine significantly lower than BMDs of patients with normal values of TRAP5b.

## Conclusions

The primary cause of low BMD in AIS was increased bone resorption.

